# Bereavement Care Experiences of Mothers Following Stillbirth and Neonatal Death: A Latent Class Analysis

**DOI:** 10.1111/1471-0528.70190

**Published:** 2026-02-24

**Authors:** Ana Nelia Jumamil, Joemer Calderon Maravilla, Joycelyn Abiog Filoteo, Charlene Joy Mendoza Sta. Maria, Angelica Villanueva Felipe, Rachelle Sarmiento, Anna Liza Alfonso, Mayla Rivera, Jose Edwardo Mamaat, Arbie Diane Flores, Mary Ann Bayani, Reinalyn Lim Cardenas, Wilfredo Quijencio, Nemencio Santos, Joyce Lisa Acena, Rebecca A. Guariño, Ida Stevia Diget, Alma Trinidad Taragua, Frances M. Boyle

**Affiliations:** ^1^ Department of Psychology, Institute of Arts and Sciences Far Eastern University Manila Metro Manila Philippines; ^2^ Institute of Health Sciences and Nursing Far Eastern University Manila Metro Manila Philippines; ^3^ School of Public Health The University of Queensland Herston Queensland Australia; ^4^ Queensland Centre for Mental Health Research Wacol Queensland Australia; ^5^ Filipino Nursing Diaspora Network Brighton‐Le Sands New South Wales Australia; ^6^ Far Eastern University Manila Metro Manila Philippines; ^7^ JPMC College of Health Sciences Bandar Seri Begawan Brunei Darussalam; ^8^ NHMRC Centre of Research Excellence in Stillbirth, Mater Research Institute The University of Queensland South Brisbane Queensland Australia; ^9^ James Cook University Townsville Queensland Australia; ^10^ Institute for Social Science Research The University of Queensland Indooroopilly Queensland Australia

**Keywords:** desired care, latent class analysis, perinatal bereavement care, Philippines, unmet need

## Abstract

**Objective:**

This study examined nuanced preferences and unmet needs for bereavement care of mothers who experienced stillbirth or neonatal death.

**Design:**

Cross‐sectional survey.

**Setting:**

The Philippines.

**Sample:**

A total of 169 bereaved mothers aged 18 years or older who had experienced stillbirth or neonatal death on or after 30 January 2020 in the Philippines.

**Methods:**

Response patterns to nine self‐administered questions about desired bereavement care and the care they received were analysed using latent class analysis. Measurement invariance was assessed across participant types and urbanicity. Multinomial logistic regression was used to examine the associations between latent classes, demographics and perinatal loss information.

**Main Outcome Measures:**

Desired type of bereavement care and unmet need for bereavement care.

**Results:**

We identified three mutually exclusive stable latent classes related to desired care and unmet needs. For desired bereavement care, one class was characterised by a strong preference for seeing, holding, spending time with, and naming the baby, but low desire in memory making (25.1%). This preference was more common among mothers from urban areas (aOR = 3.04) and those who experienced stillbirth (aOR = 3.07), compared to those from rural areas and who experienced neonatal death, respectively. Regarding unmet need for bereavement care, one class reported unmet needs for memory making (28.3%), with preterm mothers (aOR = 3.11) three times more likely to belong to this class than those who delivered full‐term.

**Conclusion:**

This study offers novel insights into the complex bereavement care preferences and needs of mothers who experienced perinatal loss. The findings can inform future frameworks and guide the development of culturally appropriate, context‐specific interventions for mothers and families.

## 1 Introduction

The loss of a baby due to stillbirth or neonatal death (NND) can profoundly impact mothers' physical, mental and psychosocial health and wellbeing. Intense emotional distress is common soon after the loss and may be enduring for some [1, 2]. Understanding the complex interplay of factors that influence the recovery process for mothers following perinatal loss is essential for informing practices that promote the wellbeing of bereaved families.

The World Health Organization defines stillbirth as the death of a baby of at least 28 weeks' gestation before or during birth [3]. Neonatal death is the death of a baby within the first 28 days of life [4]. In 2022, there were an estimated 1.9 million stillbirths worldwide, about one every 17 s, with Sub‐Saharan Africa and South Asia contributing to over 75% of global stillbirths [5, 6, 7]. In the same year, 2.3 million NNDs were reported, with Sub‐Saharan Africa experiencing the highest neonatal mortality rate at 27 per 1000 live births, followed by Central and Southern Asia at 22 per 1000 live births [3].

Perinatal bereavement care ensures that mothers receive emotional, psychological and social support [8], helping to reduce the risk of mental health complications such as prolonged grief, depression and anxiety [9, 10]. Although internationally recognised frameworks for bereavement care exist [11], they often fail to fully address cultural nuances, particularly in low‐ and middle‐income countries (LMICs), highlighting the importance of local guidelines that ensure culturally sensitive and accessible care practices.

During the COVID‐19 pandemic, the Australian Centre of Research Excellence in Stillbirth conducted an international, cross‐sectional online survey of parents who have accessed maternity, neonatal and/or bereavement care services called the COCOON Global Study [12]. This survey is co‐designed with partner organisations across 15 countries, including four LMICs. Although the COCOON Global Study sought to investigate the potential impacts of COVID‐19 on parents' experiences of these services, this study also explored ongoing underrepresented perinatal health issues such as bereavement care.

This study aimed to explore the bereavement care needs of mothers following stillbirth or neonatal death, focusing on their expressed desires for care and unmet needs. Recognising that bereaved mothers may share overlapping preferences and experiences, the study employed a person‐centred analytical approach called latent class analysis (LCA) to identify distinct patterns across different types of care. By profiling these nuanced patterns, the study provides valuable insights into the complexities of perinatal bereavement care and contributes to the development of future theoretical frameworks.

## 2 Methods

### 2.1 Design and Participants

Far Eastern University‐Manila leads the COCOON Study in the Philippines, which is an archipelagic nation comprising over 7000 islands. The Philippines is nearly evenly divided between urban and rural areas [13], with Catholicism as the predominant religion, followed by Protestantism and Islam [14]. The healthcare system is structured into three tiers: primary care delivered through barangay health stations and rural health units; secondary care provided by municipal and district hospitals; and tertiary care concentrated in regional and national facilities equipped for advanced diagnostics and specialist services.

The Philippines was chosen to be part of this study due to its relatively high stillbirth and neonatal mortality rates compared to other countries in the ASEAN and the Pacific region [15]. In 2022, the stillbirth and neonatal mortality rates in the Philippines were estimated to be at 12 per 1000 pregnancies of at least 28 weeks in duration and 15 per 1000 live births [16]. Moreover, bereavement care in the Philippines remains underdeveloped, with no specific programmes and guidelines dedicated to supporting families after stillbirth or NND.

Aligned with the global protocol of the COCOON Study [12], a descriptive survey design was employed to assess bereavement and follow‐up care received by Filipino mothers who experienced stillbirth or NND. Mothers who had experienced stillbirth or NND on or after 30 January 2020 were eligible to participate in the survey. Mothers who experienced multiple perinatal losses during this period were asked to refer to their most recent loss. The recruitment process predominantly utilised Facebook advertisements to effectively reach participants. Paid advertisements were deployed once a week for 6 weeks between March 2024 and April 2024. The face‐to‐face recruitment in birthing clinics and community health centres did not reach as many participants (see Figure S1), which may be related to stigma related to perinatal loss.

### 2.2 Survey Procedure

The online questionnaire was self‐administered and included items related to mothers' pregnancy, bereavement and postpartum care experiences, obstetric history, mental health and socio‐demographic characteristics [12]. The information sheet, consent form and questionnaire were available in both English and Tagalog‐English. The questionnaire was translated into Tagalog‐English and pilot tested.

To ensure the integrity of online participation, the research team verified potential participants through Meta Business chat to prevent bots and fraudulent accounts from participation. Verification involved collecting key details including the mother's first name, last name, date of birth, gestational age and the baby's date of birth. Only individuals who passed this verification process were provided with the survey link. Responses were further validated by checking the survey completion time and for any discrepancies between the babies' dates of birth reported during initial verification and those reported in the survey. A total of 30 responses were removed after data validation.

At the end of the survey, participants were asked to voluntarily provide their contact details for a follow‐up survey and an in‐depth interview (results are outside the scope of this study). Identifying information was securely stored separately from survey responses. Participants were provided with information about local and online support services. Two participants reported distress after the survey and were assisted by the study psychologist online via Meta Business chat and phone call, respectively.

### 2.3 Measures

#### 2.3.1 Bereavement Care

Mothers were asked nine questions to describe their experiences of bereavement care after they delivered their baby (i.e., stillbirth) or when their newborn died, as outlined in Table S1. These questions have been widely used to assess bereavement care in 40 other countries [17, 18, 19] and are aligned with bereavement care guidelines by the International Stillbirth Alliance, as well as the Australian Centre of Research Excellence in Stillbirth and the Perinatal Society of Australia and New Zealand [11, 20]. Options assessed the receiving and preference of such care with the following options: *Yes, and I'm glad I had the opportunity*; *Yes, but I did not want the opportunity*; *No, but I would have liked the opportunity*; *No, but I did not want the opportunity*; and *Prefer not to answer*.

#### 2.3.2 Perinatal Loss Information

Participants were asked about gestational age in weeks when they gave birth to their baby, which was used to determine prematurity. Maternal age was calculated using their date of birth and date of delivery. The place of death of their baby was determined by referring to the place of delivery among those who experienced stillbirth or the location of NND. The time elapsed since death in months was calculated by comparing the date of delivery with the date of the survey.

#### 2.3.3 Socio‐Demographic Characteristics

Participants were asked to report their highest level of education and marital status. Urbanicity was determined based on the municipality, city, or province they provided, using classifications established by the Philippine Statistics Authority [21].

### 2.4 Data Analysis

All analyses were conducted using Stata18.0 [22] and the LCA Stata Plug‐in v. 1.2.1 [23]. Desire for bereavement care items was assessed by grouping the responses into two [i.e., (1) I'm glad I had the opportunity/I would have liked the opportunity, (2) I did not want the opportunity/prefer not to say]. The extent of unmet need for bereavement care items was determined by obtaining the proportion of those who answered ‘No, but I would have liked the opportunity’. All participants completed the bereavement care items. Since this study primarily examines the presence of desire for care and unmet need for care, the ‘Prefer not to say’ responses were combined with other categories, as these individuals did not explicitly report a desire or unmet need for care. The distribution of non‐response for the covariates is shown in Table S2. The number of care items desired and unmet was separately calculated.

#### 2.4.1 Identification of Latent Classes of Desired and Unmet Needs for Bereavement Care

Latent class analysis (LCA) was conducted to further describe nuanced patterns in bereaved mothers' desires and unmet needs for care. This analytical approach has been known to be effective in describing nuanced patterns of mental health support [24]. LCA qualitatively describes heterogeneity within the sample by identifying subgroups or latent classes based on shared characteristics (i.e., bereavement care items in this study). Latent classes are then described using item‐response probabilities (i.e., probabilities of reporting each bereavement care item).

Two‐class to seven‐class models were generated separately for desired bereavement care and unmet needs for care based on all nine bereavement care items using maximum likelihood and expectation‐maximisation algorithms. Missing data were assumed as missing at random and were included using full information maximum likelihood estimation [25]. Posterior probabilities (or latent class membership or prevalence) and item‐response probabilities were calculated for each model.

To determine the optimal number of latent classes (or model solution), we referred to multiple fit statistics including entropy, Bayesian information criterion (BIC), sample‐size‐adjusted Bayesian information criterion (aBIC), Akaike information criterion (AIC), bootstrapped likelihood ratio test (BLRT) and sample class size. A better fit was assessed by identifying an entropy of 0.80 or higher, a lower AID and a/BIC, and a class size of > 5%. AIC and BIC are commonly used to determine model fit in studies with sample > 300 [26]. BLRT was used to test if the current class model (*k*) is a statistically better fit than the *k* − 1 class model; if *p* > 0.05, then *k* − 1 model was chosen. The interpretability of the classes was also considered during model selection. The model solution was further tested for measurement invariance by participant type (i.e., those who reported stillbirth and NND) and urbanicity using the likelihood‐ratio test (*G* ^2^Δ).

#### 2.4.2 Test of Association

The association between latent classes and pregnancy loss information, as well as socio‐demographic characteristics, was tested using multivariate multinomial logistic regression. Covariates were dichotomised as per the requirements of the LCA software plug‐in [23]. Unadjusted and adjusted odds ratios (aORs) and 95% confidence intervals (CIs) were calculated for each covariate. Only those with complete data for all covariates were included in the regression analysis.

## 3 Results

### 3.1 Sample Characteristics

A total of 169 Filipino mothers participated in the survey, comprising 111 who had experienced stillbirth and 58 who had experienced NND, with response rates of 43.2% and 47.5%, respectively. 59.8% of mothers who experienced stillbirth and neonatal death had lost their babies in the past 0–36 months to the survey, and 68.5% were non‐primiparous. A large proportion of these losses were preterm births, noting that a fifth of those with stillbirth lost their babies at full term. The majority were aged 25–34, with at least an undergraduate degree, resided in urban areas, and were living with their partner/husband (Table 1).

**TABLE 1:** Sample characteristics.

	Combined sample *n* (%)	Mothers who experienced stillbirth *n* (%)	Mothers who experienced neonatal death *n* (%)
*N*	169	111	58
Number of months since the death of the baby			
0–12 months	38 (22.5%)	21 (18.9%)	17 (29.3%)
12–36 months	63 (37.3%)	46 (41.4%)	17 (29.3%)
37+ months	68 (40.2%)	44 (39.6%)	24 (41.4%)
Prematurity			
Full‐term	46 (27.2%)	20 (18.0%)	26 (44.8%)
Preterm	123 (72.8%)	91 (82.0%)	32 (55.2%)
Place of death			
At home	30 (17.8%)	15 (13.5%)	15 (25.9%)
Birthing clinic or health centre	21 (12.4%)	18 (16.2%)	3 (5.2%)
Hospital	118 (69.8%)	78 (70.3%)	40 (69.0%)
First pregnancy^a^			
Yes	52 (31.5%)	35 (32.4%)	17 (29.8%)
No	113 (68.5%)	73 (67.6%)	40 (70.2%)
Maternal age			
< 18–24 years old	54 (32.0%)	38 (34.2%)	16 (27.6%)
25–34 years old	103 (60.9%)	65 (58.6%)	38 (65.5%)
35–44 years old	12 (7.1%)	8 (7.2%)	4 (6.9%)
Highest educational attainment^a^			
Secondary or lower	74 (45.7%)	49 (46.7%)	25 (43.9%)
University or higher	88 (54.3%)	56 (53.3%)	32 (56.1%)
Relationship status^a^			
No partner/not living with partner	19 (11.5%)	15 (13.8%)	4 (7.1%)
Living with partner	146 (88.5%)	94 (86.2%)	52 (92.9%)
Urbanicity			
Rural	44 (26.0%)	29 (26.1%)	15 (25.9%)
Urban	125 (74.0%)	82 (73.9%)	43 (74.1%)

^a^ The denominator (*N*) was different due to non‐response for the following variables: First pregnancy = 165 (stillbirth: 108, neonatal death: 57); highest educational attainment = 162 (stillbirth: 105, neonatal death: 57); relationship status = 165 (stillbirth: 109, neonatal death: 56). See Table S2 for the distribution of non‐response on these variables.

### 3.2 Desired Bereavement Care

The item‐level analysis in Table S3 shows that > 70% of mothers wished to receive bereavement care based on all nine items. No statistical differences in individual item preference were observed between mothers who experienced stillbirth and NND.

Using a progressive model selection approach in the identification of latent classes, BIC, aBIC, AIC, entropy and BLRT of the three‐class model outperformed the four‐class model (see Table S4). BLRT showed that there was no significant difference between these models (*p* = 0.97). BIC comparison of two‐ and three‐class models showed the former; however, aBIC, AIC, entropy and BLRT showed a better fit in the three‐class model. These fit statistics and the interpretability of latent classes from the two‐, three‐ and four‐class models revealed a three‐class model solution as the most suitable for desired bereavement care. The three‐class model showed measurement invariance by participant type (*G* ^2^Δ = 24.83, df = 27, *p* = 0.58) and urbanicity (*G* ^2^Δ = 27.79, df = 27, *p* = 0.42).

**FIGURE 1:**
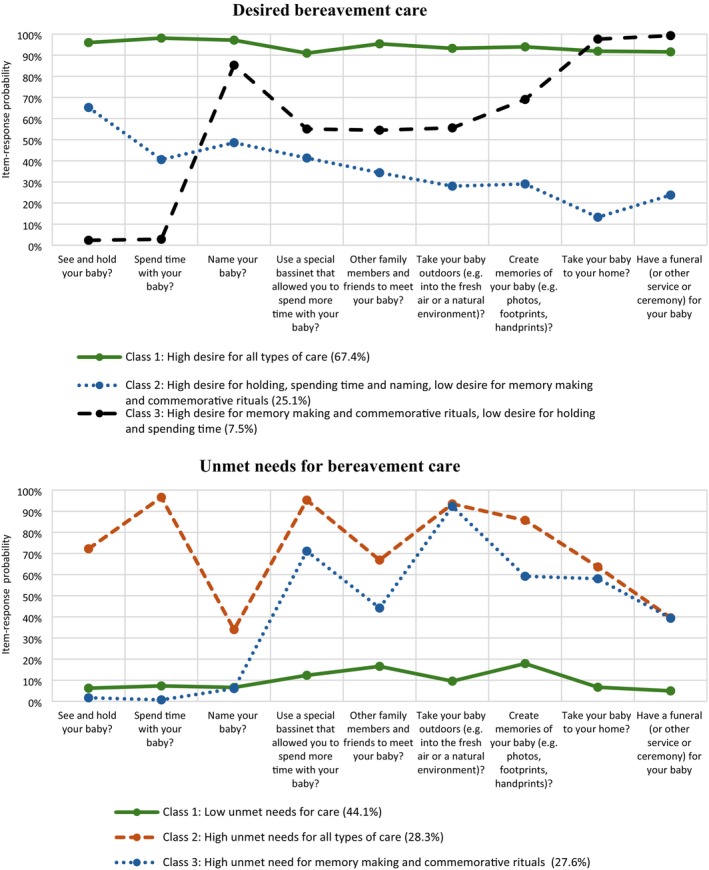
Item‐response probabilities for desired and unmet needs for bereavement care in the three‐class model solution based on latent class analysis. *Note:* Posterior probabilities or latent class memberships are presented in the graph legends.

Figure 1 illustrates the three latent classes, each representing a distinct pattern of desired care. *The first latent class* showed high probabilities (> 90.0%) of desiring all nine care items, with the largest class membership (or prevalence) of 67.4% (*n* = 114). A quarter (25.1%, *n* = 42) of bereaved mothers belonged to *the second latent class* which demonstrated high desire for seeing and holding (65.3%), spending time (40.6%) and naming (48.6%) their baby, but low desire for taking their baby home (13.3%), memory making (29.0%) and funeral (23.8%). *The third latent class* reported a high desire for memory making (69.0%) and commemorative rituals such as taking their baby home (97.6%) and having a funeral service (99.3%) but had low desire to see and hold (2.4%) and spend time (2.9%) with their baby. This has a class membership of 7.5% (*n* = 13).

Given the small cell size due to small Class 3 membership, we used a stepwise modelling approach including only covariates with statistically significant unadjusted ORs in the adjusted model to minimise bias from overfitting. The analysis revealed differences in latent class membership by participant type and urbanicity (see Table 2, Table S5 for full regression results). Thrice as many mothers who experienced stillbirth (aOR = 3.04, 95% CI = 1.28–7.21) and those from urban areas (aOR = 3.07, 95% CI = 1.13–8.35) were three times as likely to prefer a specific type of care (i.e., Class 2: High desire for holding, spending time and naming, low desire for memory making and commemorative rituals) compared to those who experienced NND and from rural areas, respectively.

**TABLE 2:** Unadjusted and adjusted odds ratios (95% confidence interval) of desired and unmet needs for bereavement care based on the three‐class model solution.

Associated covariates	Unadjusted	Adjusted	Unadjusted	Adjusted
	Class 2 membership		Class 3 membership	
Desired bereavement care^a^				
Participant type				
Mothers who experienced stillbirth (ref. neonatal death)	2.94 (1.24–6.96)	3.04 (1.28–7.21)	2.56 (0.60–10.84)	2.65 (0.64–11.01)
First pregnancy				
Yes (ref. no)	2.22 (1.02–4.82)	2.13 (0.97–4.69)	2.35 (0.69–8.00)	2.45 (0.73–8.23)
Urbanicity				
Urban (ref. rural)	3.24 (1.17–8.95)	3.07 (1.13–8.35)	0.98 (0.27–3.53)	0.98 (0.27–3.54)
Unmet needs for bereavement care^b^				
Prematurity				
Preterm (ref. full‐term)	1.61 (0.72–3.62)	1.26 (0.63–2.52)	4.42 (1.37–14.26)	3.11 (1.29–7.50)

*Note:* Only shows associated covariates. Please refer to Tables S5 and S6 for full regression results.

^a^ Class 1: High desire for all types of care (reference outcome); Class 2 High desire for holding, spending time and naming, low interest for memory making and commemorative rituals; Class 3: High desire for memory making and commemorative rituals, low interest for holding and spending time. The adjusted odds ratio accounted for participant type, first pregnancy and urbanicity. Non‐response data on the first‐pregnancy covariate reduced the sample size for adjusted analyses to *N* = 165 (Class 2 vs. Class 1 *n* = 152; Class 3 vs. Class 1 *n* = 55).

^b^ Class 1: Low unmet needs for care (reference outcome); Class 2: High unmet needs for all types of care; Class 3: High unmet need for memory making and commemorative rituals. The adjusted odds ratio accounted for all covariates. Non‐response data on the first pregnancy, educational attainment and marital status covariate reduced the sample size for adjusted analyses to *N* = 157 (Class 2 vs. Class 1 *n* = 117; Class 3 vs. Class 1 *n* = 109).

### 3.3 Unmet Needs for Bereavement Care

As shown in Table S3, approximately 50% of bereaved mothers reported unmet need for memory making (48.5%), use of a bassinet to spend time with their baby (52.1%) and taking the baby outdoors (56.2%). Less than 30% were found with unmet need for naming (14.2%), seeing and holding (23.7%) and holding a funeral or ceremony for the baby (24.3%). No statistical differences were noted between participant types.

The three‐class model solution for unmet needs for bereavement care was supported by fit statistics (see Table S4). BIC and SCS suggested three classes, while aBIC, AIC and entropy were similar between the three‐ and four‐class models. BLRT further indicated three classes as the model of choice (*p* = 0.43). The three‐class model showed measurement invariance by participant type (*G* ^2^Δ = 30.57, df = 27, *p* = 0.29) and urbanicity (*G* ^2^Δ = 20.55, df = 27, *p* = 0.81).

As shown in Figure 1, the first latent class included mothers with low probabilities of unmet needs across all care items (< 20.0%). The second class demonstrated higher probabilities of unmet needs in all care items compared to other latent classes. The third class showed high unmet needs for memory making (59.2%) and commemorative rituals such as spending time with their baby in natural environments (92.3%), taking their baby home (58.1%) and having a funeral service (39.3%). Class memberships are 44.1% (*n* = 74), 28.3% (*n* = 48) and 27.6% (*n* = 47), respectively.

The adjusted regression analysis for unmet need for care included all covariates due to adequate class membership and cell sizes. A difference in class membership was observed by prematurity, as those with preterm babies were three times more likely to report unmet need for memory making and commemorative rituals (Class 3 aOR = 3.11, 95% CI = 1.29–7.50) than those with full‐term babies (see Table 2, Table S6 for full regression results).

## 4 Discussion

### 4.1 Main Findings

This study provides nuanced information about the desire for bereavement care and unmet need for care of mothers who have experienced stillbirth or NND. Two out of three bereaved mothers expressed a strong desire for comprehensive care (*Class 1 based on LCA of desired care*) including spending time with their baby, naming them and other forms of emotional support. One in four bereaved mothers (*Class 2*) placed a high priority on holding and spending time with their baby, but showed a lower desire for memory‐making and commemorative rituals. One in every 13 (*Class 3*) had the highest desire for creating lasting memories and engaging in commemorative rituals, while their interest in holding or spending time with their baby is minimal. In terms of unmet need for care, four out of ten bereaved mothers showed low unmet needs across all care types (*Class 1 based on LCA of unmet need for care*). Six out of ten reported forms of unmet need, either with all types of care (*Class 2*) or with memory‐making and commemorative rituals (*Class* 3).

### 4.2 Interpretation

The distinct patterns in the desired type of bereavement care identified by our study underscore the importance of context‐specific approaches and culturally sensitive interventions. A cross‐national survey of parents of stillborn babies revealed differences in bereavement care preferences between high‐income (HICs) and middle‐income countries (MICs) [18]. More parents in MICs preferred to take their babies home compared to those in HICs, with 64.5% expressing this preference, which closely aligns with our study's findings. Parents in MICs were less likely to report wanting to spend time with their baby (82.4%), see and hold their baby (82.1%) or have a funeral or ceremony (78.1%). Our study showed slightly lower proportions: 72.1%, 77.5% and 72.1%, respectively.

Apart from nuanced patterns of preference in care, we also found differences in preferences based on certain characteristics of bereaved mothers. For example, we found that mothers who experience stillbirth and those from urban areas are thrice as likely to prefer direct interaction, including holding, naming and spending time with their baby, compared to those who experienced neonatal loss and those from rural areas. Identifying potential factors influencing preferential differences between mothers from urban and rural areas such as greater reliance on community‐based mourning practices among mothers from rural areas [27], is essential for informing public health strategies supporting bereaved mothers. The nuanced understanding of bereavement care generated by our study underscores the need for diverse types of care and provision of personalised support, allowing mothers to navigate grief in a way that aligns with their emotional and cultural needs.

Our findings revealed a higher prevalence of unmet need in memory‐making activities (55.9%, combined prevalence of Class 2 and Class 3) compared to the overall rate reported in MICs (49.5%) [18]. Implementing best practices in memory‐making has been shown to positively influence maternal mental health and reduce feelings of profound regret [28, 29]. Although memory‐making may initially evoke intense grief—which can cause hesitation among healthcare workers to offer such care, many bereaved parents have expressed deep appreciation for these practices over time, noting their role in preserving a sense of parenthood and facilitating emotional healing [28, 30, 31]. A common challenge reported in healthcare settings is the lack of institutional policies and protocols to support memory‐making activities [28, 32]. These include inadequate staff preparation, limited time between delivery and the aftercare arrangements for the deceased baby, and insufficient resources or training to guide families through the process. Addressing these gaps through culturally responsive care models, policy reform and workforce education is critical to improving bereavement support for families.

The unmet need revealed in our study was generally lower compared to other MICs [18]. This can be attributed to several context‐specific factors. In the Philippine context, naming a stillborn or neonatal death is a legal requirement and is closely tied to birth and death registration processes [33]. Naming fosters a deep emotional connection between parents and their unborn child and reflects the importance of family and identity in Filipino culture [34]. Filipino mourning practices emphasise stronger family involvement, spiritual and religious elements, and community‐based support rather than formal psychological interventions [35]. Although Western models focus on individual therapy and structured counselling [36], Filipino families often rely on faith‐based healing and collective mourning rituals [35].

Given these cultural distinctions, healthcare providers should integrate spiritual and cultural elements into bereavement care, encourage family‐centred grief support, and acknowledge the significance of continuing bonds in mourning traditions. The establishment of community‐based support services, such as peer groups and accessible counselling services, will provide sustained practical and emotional support within local communities. Public awareness campaigns can also help break the stigma surrounding stillbirth and NND, fostering a more supportive environment where families feel encouraged to seek help without fear of judgment [37].

The high proportion of unmet care needs identified in our study reflects the lack of guidelines in the country [11, 38]. Developing bereavement care guidelines in culturally diverse settings, such as the Philippines, requires a comprehensive and consultative process to ensure they are both evidence‐based and deeply rooted in local cultural values and practices. Collaboration among cultural experts, healthcare providers, and grieving families is crucial in crafting care protocols that align with local customs while fostering emotional healing. Integrating traditional practices and faith‐based rituals into these guidelines can help ensure that bereavement support is meaningful, respectful and culturally relevant [39].

Poor communication and a lack of empathy from healthcare providers can deter mothers from seeking emotional support. Enhancing competencies in compassionate communication and cultural sensitivity enables care providers to deliver holistic, empathetic care that better addresses the emotional and cultural needs of mothers and their families [36, 37]. It is also essential to equip care providers with strategies to safeguard their own mental and emotional wellbeing when delivering bereavement care. Collaborating with the higher education sector to integrate bereavement care into health curricula may accelerate its recognition as a core component of maternal care, fostering its adoption as a standard practice.

Continuous research into the experiences of bereaved families from culturally diverse backgrounds and high‐burden countries is crucial for refining global and local care guidance [40, 41]. Studies that explore the socio‐cultural and systemic factors influencing bereavement care can provide valuable insights for developing effective person‐centred interventions. Findings from such research should inform ongoing improvements to care guidelines, ensuring they remain relevant to the needs of bereaved families.

### 4.3 Strengths and Limitations

This study pioneered the identification of distinct patterns of overlapping bereavement care preferences and unmet needs among bereaved mothers, moving beyond the traditional approach of examining bereavement care types in isolation. It also successfully recruited a substantial sample of bereaved mothers despite the stigma surrounding perinatal loss. As such, our study offers foundational quantitative evidence on perinatal bereavement care in high‐burden settings such as the Philippines.

This study also has limitations. First, participants were recruited online using non‐probability sampling, which may limit the generalisability of findings to the national population. Nonetheless, this approach was deemed appropriate given the prevailing stigma surrounding perinatal loss in the Philippines, which may hinder participation through traditional recruitment methods. Moreover, the study sample primarily comprised mothers aged 20–29 years, with tertiary education, and residing in urban areas. This demographic profile differs from national statistics in the Philippines, where stillbirth and neonatal mortality rates are disproportionately higher among teenage mothers, those without tertiary education, and those living in rural areas (see Table S7). Although the characteristics of our sample are not fully aligned with national statistics, measurement invariance testing indicated that the latent classes identified in this study were robust across mothers residing in both rural and urban settings. Second, results from the LCA should be interpreted with caution due to the small sample size of this study; nonetheless, multiple fit statistics were used to provide a thorough approach to selecting the optimal class model, and invariance testing was conducted to ensure the robustness of the model solution. Third, correlation estimates, particularly in multivariable models, should also be interpreted with caution due to small cell sizes. Fourth, the survey items used to assess bereavement care experiences may not have fully captured context‐specific care needs, as no validated tool currently exists in the country for this purpose. To address these gaps, in‐depth qualitative investigations are essential for confirming nuanced patterns observed in this study and for exploring specific care needs that can inform future guidelines and interventions. Our research team has completed in‐depth interviews with a nested sample of mothers from the survey, and analysis is currently underway. Findings from this qualitative research will be reported separately to provide a comprehensive account of bereavement care preferences and experiences within the Philippine context.

## 5 Conclusion

Bereaved mothers expressed a strong desire for comprehensive and specific types of bereavement care; however, most did not receive the care they preferred. This highlights the urgent need for context‐specific bereavement care interventions to support mothers and families. Urban–rural differences in care preferences further underscore the importance of community‐based support systems including culturally rooted mourning practices. In a culturally diverse setting with a complex policy landscape such as the Philippines, integrating bereavement care in basic maternal care and developing culturally responsive care guidelines is essential to ensure that the needs of mothers who have experienced perinatal loss are adequately met, thereby supporting their journey toward healing and recovery.

## Author Contributions

A.N.J., J.C.M. and J.A.F. conceptualised the study, while F.M.B. developed the original methods for the COCOON Global Study. A.N.J., J.C.M., J.A.F., M.A.B., R.L.C. and W.Q. developed the survey methods in the Philippines. A.N.J., J.C.M., J.A.F., R.S., A.L.A., M.R., J.E.M., A.D.F., M.A.B., R.L.C., W.Q., N.S.J., J.L.A. and A.T. conducted participant screening and online data collection, while J.A.F., A.L.A., W.Q. and J.L.A. conducted the survey face‐to‐face. A.N.J., J.C.M. and C.J.M.S.M. performed the formal analysis, while A.N.J., J.C.M., J.A.F., C.J.M.S.M. and A.V.F. completed the data validation. A.N.J., J.C.M. and J.A.F. wrote the original version of the manuscript, while all other authors contributed to the reviewing and revision of the manuscript.

## Funding

The core funding of the COCOON Study in the Philippines is provided by Far Eastern University, Manila, Philippines. Dr. Joemer Maravilla is supported by the Centre of Research Excellence in Adolescent Health (APP1171981), which receives funding from the National Health and Medical Research Council (NHMRC) in Australia. The research reported in this publication is part of the Centre of Research Excellence in Stillbirth (Stillbirth CRE). Core funding to support the Stillbirth CRE is provided by the NHMRC. We also acknowledge the support provided by the Mater Foundation.

## Ethics Statement

Ethics approval for the multicounty survey has been granted by the Mater Misericordiae Human Research Ethics Committee in Australia (reference number: AM/MML/63526). This study is also approved by the Far Eastern University Ethics Review Committee (2023–2024‐008) for local data collection in the Philippines. All participants provided informed consent, and data were further deidentified.

## Conflicts of Interest

The authors declared no competing interests.

## Supporting information


**Table S1:** Bereavement care items.
**Table S2:** Distribution of non‐response.
**Table S3:** Prevalence of desire and unmet need for bereavement care by item.
**Table S4:** Model fit statistics of latent class models for desired bereavement care and unmet needs for bereavement care.
**Table S5:** Unadjusted and adjusted odds ratios (95% confidence interval) of desire for a specific type of bereavement care.
**Table S6:** Unadjusted and adjusted odds ratios (95% confidence interval) of unmet needs for bereavement care.
**Table S7:** Characteristics of the survey participants and the patterns of stillbirth and neonatal mortality rates in the Philippines.
**Figure S1:** Participant selection flowchart.

## Data Availability

The data that support the findings of this study are available from the corresponding author upon reasonable request.
