# Supplementary material for: Bereavement Care Experiences of Mothers Following Stillbirth and Neonatal Death: A Latent Class Analysis
Source: BJOG. 2026 Feb 24;133(7):1465–74. doi: 10.1111/1471-0528.70190 (PMC13143561; doi:10.1111/1471-0528.70190)
Supplement: Supplementary file 1 — Table S1: Bereavement care items. Table S2: Distribution of non‐response. Table S3: Prevalence of desire and unmet need for bereavement care by item. Table S4: Model fit statistics of latent class models for desired bereavement care and unmet needs for bereavement care. Table S5: Unadjusted and adjusted odds ratios (95% confidence interval) of desire for a specific type of bereavement care. Table S6: Unadjusted and adjusted odds ratios (95% confidence interval) of unmet needs for bereavement care. Table S7: Characteristics of the survey participants and the patterns of stillbirth and neonatal mortality rates in the Philippines. Figure S1: Participant selection flowchart. [file BJO-133-1465-s001.docx]

Contents

[**Table S1. Bereavement care items** 2](#_Toc219929930)

[**Table S2. Distribution of non-response** 3](#_Toc219929931)

[**Table S3. Prevalence of desire and unmet need for bereavement care by item** 4](#_Toc219929932)

[**Table S4. Model fit statistics of latent class models for desired bereavement care and unmet needs for bereavement care** 5](#_Toc219929933)

[**Table S5. Unadjusted and adjusted odds ratios (95% confidence interval) of desire for a specific type of bereavement care** 6](#_Toc219929934)

[**Table S6. Unadjusted and adjusted odds ratios (95% confidence interval) of unmet needs for bereavement care** 7](#_Toc219929935)

[**Table S7. Characteristics of the survey participants and the patterns of stillbirth and neonatal mortality rates in the Philippines** 8](#_Toc219929936)

[**Figure S1. Participant selection flowchart** 9](#_Toc219929937)

# **Table S1. Bereavement care items**

| **English** | **Tagalog-English** |
| --- | --- |
| After your baby died, were you given the opportunity by your care provider to:   1. See and hold your baby? 2. Spend time with your baby? 3. Other family members and friends to meet your baby? 4. Name your baby? 5. Create memories of your baby (e.g. photos, footprints, handprints)? 6. Use a special bassinet (with a small cooling unit inside) that allows you to spend more time with your baby? 7. Take your baby outdoors (e.g. into the fresh air or a natural environment)? 8. Take your baby to your home? 9. Have a funeral (or other service or ceremony) for your baby? | Matapos mamatay ang iyong sanggol, binigyan ka ba ng pagkakataon ng iyong tagapagbigay ng pangangalaga o health worker:   1. Makita at mahawakan ang iyong sanggol? 2. Gumugol ng oras kasama ang iyong sanggol? 3. Makilala ng iba pang miyembro ng pamilya at kaibigan ang iyong sanggol? 4. Pangalanan ang iyong sanggol? 5. Lumikha ng mga alaala ng iyong sanggol (Halimbawa,mga larawan, footprint, handprint)? 6. Gumamit ng espesyal na bassinet (na may maliit na cooling unit sa loob) na nagpapahintulot sa iyo na gumugol ng mas maraming oras kasama ang iyong sanggol? 7. Dalahin ang iyong sanggol sa labas (Halimbawa, sa sariwang hangin o natural na kapaligiran)? 8. Dalahin ang iyong sanggol sa iyong tahanan? 9. Magkaroon ng libing (o iba pang seremonya) para sa iyong sanggol? |

# **Table S2. Distribution of non-response**

| **Participant characteristics variables** | **Combined sample**  **n** | **Mothers who experienced stillbirth**  **n** | **Mothers who experienced neonatal death**  **n** |
| --- | --- | --- | --- |
| Number of months since the death of the baby | 0 | - | - |
| Prematurity | 0 | - | - |
| Place of death | 0 | - | - |
| First pregnancy | 4 | 3 | 1 |
| Maternal age | 0 | - | - |
| Highest educational attainment | 7 | 6 | 1 |
| Relationship status | 4 | 2 | 2 |
| Urbanicity | 0 | - | - |

# **Table S3. Prevalence of desire and unmet need for bereavement care by item**

| **Item** | **Desire for care** | | | **Unmet need for care** | | |
| --- | --- | --- | --- | --- | --- | --- |
|  | **Combined** | **Stillbirth** | **NND** | **Combined** | **Stillbirth** | **NND** |
| See and hold your baby? | 81.1% | 77.5% | 87.9% | 23.7% | 27.9% | 15.5% |
| Spend time with your baby? | 76.3% | 72.1% | 84.5% | 30.8% | 35.1% | 22.4% |
| Name your baby? | 84.0% | 80.2% | 91.4% | 14.2% | 15.3% | 12.1% |
| Use a special bassinet (with a small cooling unit inside) that allows you to spend more time with your baby? | 75.7% | 73.0% | 81.0% | 52.1% | 55.9% | 44.8% |
| Other family members and friends to meet your baby? | 76.9% | 73.0% | 84.5% | 38.5% | 36.9% | 41.4% |
| Take your baby outdoors (e.g. into the fresh air or a natural environment)? | 74.0% | 69.4% | 82.8% | 56.2% | 55.9% | 56.9% |
| Create memories of your baby (e.g. photos, footprints, handprints)? | 75.7% | 70.3% | 86.2% | 48.5% | 49.5% | 46.6% |
| Take your baby to your home?* | 71.9% | 67.7% | 81.4% | 38.1% | 32.3% | 51.2% |
| Have a funeral (or other service or ceremony) for your baby? | 75.1% | 72.1% | 81.0% | 24.3% | 25.2% | 22.4% |

Notes: Stillbirth, mothers who experienced stillbirth; NND, mothers who experienced neonatal death; None were statistically different at p<0.05; *Excludes those whose baby died at home [stillbirth (n=15); neonatal death (n=15)]

# **Table S4. Model fit statistics of latent class models for desired bereavement care and unmet needs for bereavement care**

| **Number of classes** | **LL** | **Entropy** | **BIC** | **aBIC** | **AIC** | **BLRT** | **SCS** |
| --- | --- | --- | --- | --- | --- | --- | --- |
| **Desired bereavement care** |  |  |  |  |  |  |  |
| **2** | -628.17 | 0.92 | 352.58 | 292.43 | 293.12 | 0.01 | 30.40 |
| **3** | -609.01 | 0.95 | 365.57 | 273.74 | 274.80 | 0.01 | 7.53 |
| **4** | -602.74 | 0.90 | 404.33 | 280.85 | 282.27 | 0.97 | 5.42 |
| **5** | -581.83 | 0.90 | 413.80 | 258.66 | 260.44 | 0.01 | 7.15 |
| **6** | -576.91 | 0.93 | 455.27 | 268.46 | 270.61 | 0.99 | 6.23 |
| **7** | -571.20 | 0.89 | 495.15 | 276.68 | 279.19 | 0.01 | 6.07 |
| **Unmet needs for bereavement care** |  |  |  |  |  |  |  |
| **2** | -766.30 | 0.84 | 355.89 | 295.73 | 296.42 | 0.01 | 46.47 |
| **3** | -731.62 | 0.87 | 337.85 | 246.02 | 247.08 | 0.01 | 27.62 |
| **4** | -721.68 | 0.89 | 369.25 | 245.76 | 247.18 | 0.43 | 7.07 |
| **5** | -710.84 | 0.87 | 398.87 | 243.72 | 245.50 | 0.16 | 6.99 |
| **6** | -705.51 | 0.82 | 439.51 | 252.70 | 254.84 | 0.99 | 1.22 |
| **7** | -698.46 | 0.88 | 476.71 | 258.23 | 260.75 | 0.61 | 0.50 |

Notes: LL, Log-likelihood; BIC, Bayesian information criterion; aBIC, sample-size adjusted BIC; AIC, Akaike information criterion; BLRT, p-value from bootstrapped likelihood ratio test; SCS, proportion (%) of the smallest class size

# **Table S5. Unadjusted and adjusted odds ratios (95% confidence interval) of desire for a specific type of bereavement care**

| **Covariates** | **Class 2 membership** | | **Class 3 membership** | |
| --- | --- | --- | --- | --- |
|  | **Unadjusted** | **Adjusted** | **Unadjusted** | **Adjusted** |
| Participant type |  |  |  |  |
| Mothers who experienced stillbirth  (Ref. neonatal death) | **2.94**  **(1.24 - 6.96)** | **3.04**  **(1.28 - 7.21)** | 2.56  (0.60 – 10.84) | 2.65  (0.64 – 11.01) |
| Number of months since the death of the baby |  |  |  |  |
| 25+ months  (Ref. 0-24 months) | 1.60  (0.76 - 3.40) |  | 1.05  (0.31 - 3.50) |  |
| Prematurity |  |  |  |  |
| Preterm  (Ref. full-term) | 0.82  (0.32 - 2.07) |  | * |  |
| Place of death |  |  |  |  |
| Birthing clinic, health centre, or hospital  (Ref. at home) | 1.42  (0.53 - 3.77) |  | 3.78  (0.25 - 57.07) |  |
| First pregnancy |  |  |  |  |
| Yes  (Ref. no) | **2.22**  **(1.02 - 4.82)** | 2.13  (0.97 - 4.69) | 2.35  (0.69 - 8.00) | 2.45  (0.73 - 8.23) |
| Maternal age |  |  |  |  |
| 25-44 years old  (Ref. <18-24 years old) | 0.61  (0.28 - 1.31) |  | 0.96  (0.26 - 3.60) |  |
| Highest educational attainment |  |  |  |  |
| University or higher  (Ref. secondary or lower) | 1.15  (0.53 - 2.46) |  | 1.25  (0.35 - 4.54) |  |
| Relationship status |  |  |  |  |
| Living with partner  (Ref. no partner/not living with partner) | 0.61  (0.21 - 1.77) |  | 1.32  (0.15 – 11.41) |  |
| Urbanicity |  |  |  |  |
| Urban  (Ref. rural) | **3.24**  **(1.17 - 8.95)** | **3.07**  **(1.13 - 8.35)** | 0.98  (0.27 - 3.53) | 0.98  (0.27 - 3.54) |

Class 1: High desire for all types of care (reference outcome); Class 2 High desire for holding, spending time and naming, low interest for memory making and commemorative rituals; Class 3: High desire for memory making and commemorative rituals, low interest for holding and spending time. The adjusted odds ratio accounted for participant type, first pregnancy, and urbanicity. Non-response data on the first‑pregnancy covariate reduced the sample size for adjusted analyses to N=165 (Class 2 vs Class 1 n=152; Class 3 vs Class 1 n=55).

*Regression was not conducted due to a null cell size.

# **Table S6. Unadjusted and adjusted odds ratios (95% confidence interval) of unmet needs for bereavement care**

| **Covariates** | **Class 2 membership** | | **Class 3 membership** | |
| --- | --- | --- | --- | --- |
|  | **Unadjusted** | **Adjusted** | **Unadjusted** | **Adjusted** |
| Participant type |  |  |  |  |
| Mothers who experienced stillbirth  (Ref. neonatal death) | 1.55  (0.67 - 3.57) | 1.37  (0.69 - 2.73) | 0.82  (0.35 - 1.91) | 0.69  (0.33 - 1.47) |
| Number of months since the death of the baby |  |  |  |  |
| 25+ months  (Ref. 0-24 months) | 0.47  (0.21 - 1.00) | 0.65  (0.35 - 1.23) | 0.58  (0.25 - 1.37) | 0.81  (0.40 - 1.67) |
| Prematurity |  |  |  |  |
| Preterm  (Ref. full-term) | 1.61  (0.72 - 3.62) | 1.26  (0.63 - 2.52) | **4.42**  **(1.37 - 14.26)** | **3.11**  **(1.29 - 7.50)** |
| Place of death |  |  |  |  |
| Birthing clinic, health centre, or hospital  (Ref. at home) | 1.68  (0.63 - 4.44) | 1.48  (0.67 - 3.27) | 2.06  (0.62 - 6.91) | 1.89  (0.72 - 5.00) |
| First pregnancy |  |  |  |  |
| Yes  (Ref. no) | 0.48  (0.20 - 1.16) | 0.68  (0.32 - 1.43) | 0.80  (0.29 - 2.22) | 0.87  (0.39 - 1.96) |
| Maternal age |  |  |  |  |
| 25-44 years old  (Ref. <18-24 years old) | 1.30  (0.57 - 2.95) | 1.13  (0.54 - 2.38) | 1.17  (0.40 - 3.35) | 1.23  (0.53 - 2.89) |
| Highest educational attainment |  |  |  |  |
| University or higher  (Ref. secondary or lower) | 0.70  (0.33 - 1.50) | 0.72  (0.36 - 1.44) | 1.05  (0.44 - 2.46) | 0.79  (0.36 - 1.72) |
| Relationship status |  |  |  |  |
| Living with partner  (Ref. no partner/not living with partner) | 1.22  (0.34 - 4.35) | 1.15  (0.43 - 3.05) | 0.58  (0.17 - 1.95) | 0.82  (0.30 - 2.23) |
| Urbanicity |  |  |  |  |
| Urban  (Ref. rural) | 0.57  (0.25 - 1.30) | 0.64  (0.33 - 1.25) | 0.95  (0.36 - 2.51) | 0.84  (0.39 - 1.83) |

Note: Estimates are presented as adjusted odds ratios (95% Confidence interval). The adjusted odds ratio accounted for all covariates.

Class 1: Low unmet needs for care (reference outcome); Class 2: High unmet needs for all types of care; Class 3: High unmet need for memory making and commemorative rituals. Non-response data on the first pregnancy, educational attainment, and marital status covariate reduced the sample size for adjusted analyses to N=157 (Class 2 vs Class 1 n=117; Class 3 vs Class 1 n=109).

# **Table S7. Characteristics of the survey participants and the patterns of stillbirth and neonatal mortality rates in the Philippines**

|  | **Stillbirth** | | **Neonatal death** | |
| --- | --- | --- | --- | --- |
|  | **Distribution of participants in the survey** | **Stillbirth rate per 1000 pregnancies of 28+ weeks’ duration^[[1]](#footnote-1)^** | **Distribution of participants in the survey** | **Neonatal mortality rate per 1000 live births^1^** |
| **Maternal age** |  |  |  |  |
| <20 years old | 11.7% | 16 | 5.2% | 12 |
| 20-29 years old | 61.3% | 8 | 60.3% | 10 |
| 30+ years old | 27.0% | 15 |  |  |
| **Highest educational attainment** |  |  |  |  |
| Secondary or lower | 46.7% | 13 |  |  |
| University or higher | 53.3% | 7 |  |  |
| **Urbanicity** |  |  |  |  |
| Rural | 26.1% | 16 | 25.9% | 14 |
| Urban | 73.9% | 8 | 74.1% | 16 |

# **Figure S1. Participant selection flowchart**

**2924 reached and screened**

- 163 via face-to-face from 7 maternal care facilities
- 461 organically via Facebook (i.e., snowballed)
- 2300 via Facebook advertisements

**Postpartum women who experienced stillbirth**

266 eligible

2

via face-to-face

264

via online, provided with the survey link

**Postpartum women who experienced neonatal death**

139 eligible

0

via face-to-face

139

via online, provided with the survey link

2

113

**Started the survey**

**115 in total**

**Response rate = 43.2%**

66

**66 in total**

**Response rate = 47.5%**

2

109

(108+ 1*)

**Completed the survey**

Final sample

**111**

58

Final sample

**58**

**2519 were not eligible**

- 1224 screened as eligible for other surveys in COCOON
- 73 were not further contacted due to potentially fraudulent Facebook accounts and mismatched pregnancy information provided during the initial chat/contact, 30 reported having stillbirth (n=19) or neonatal death (n=11)
- 1222 had never been pregnant or given birth before 30 January 2020

**Partially completed the survey, at least the psychosocial wellbeing module*

**224 online participants were non-contactable after the survey link was provided**

- 151 with stillbirth
- 73 with neonatal death

Notes: The response rate was calculated by dividing the number of participants who started the survey by the number of eligible participants provided with the survey link.

1. Unweighted proportion; Source: Philippine Statistics Authority (PSA) and ICF. 2023. 2022 Philippine National Demographic and Health Survey (NDHS): Final Report. Quezon City, Philippines, and Rockville, Maryland, USA: PSA and ICF. [↑](#footnote-ref-1)
